# Broadly Neutralizing Antibodies to SARS-CoV-2 Provide Novel Insights Into the Neutralization of Variants and Other Human Coronaviruses

**DOI:** 10.3389/fcimb.2022.928279

**Published:** 2022-06-16

**Authors:** Prashant Bajpai, Vanshika Singh, Anmol Chandele, Sanjeev Kumar

**Affiliations:** ICGEB-Emory Vaccine Center, International Centre for Genetic Engineering and Biotechnology (ICGEB), New Delhi, India

**Keywords:** human coronaviruses, alphacoronaviruses, betacoronaviruses, SARS-CoV-2, broadly neutralizing antibodies, receptor binding domain, fusion peptide, stem-helix domain

## Introduction

Seven coronaviruses are known to cause infection/disease in humans. Of these, human coronavirus (HCoV)-229E and HCoV-NL63 are alphacoronaviruses (α-CoVs), whereas OC43, HKU1, Severe Acute Respiratory Syndrome Coronavirus (SARS-CoV), Middle East Respiratory Syndrome Coronavirus (MERS-CoV), and Severe Acute Respiratory Syndrome Coronavirus 2 (SARS-CoV-2) are betacoronaviruses (β-CoVs) (V’kovski et al., 2021). The recently emerged, highly mutated, and transmissible Omicron (B.1.1.529) and its variants have now become the dominant circulating SARS-CoV-2 variants worldwide with recombinant SARS-CoV-2 variants XD, XE, and XF also causing increased infection in humans (Elbe and Buckland-Merrett, 2017). Most of the concerning mutations are being reported in the spike protein of SARS-CoV-2 which is primarily responsible for the entry of the virus into the host cells, which it does through binding of its receptor-binding domain (RBD) to angiotensin-converting enzyme 2 (ACE2) receptor present on the host cells (V’kovski et al., 2021). The spike protein contains two subunits (S1 and S2) and two cleavage sites that are present at S1/S2 and S2’ sites. The S1/S2 site is cleaved by the endogenous enzyme furin whereas S2’ site is cleaved by the membrane enzyme TMPRSS2 (V’kovski et al., 2021). The S1 subunit sheds after RBD binding to ACE2 and the S2’ site is cleaved which leads to a conformational rearrangement of the S2 subunit for the insertion of fusion peptide into the host cell membrane. The spike protein is therefore a primary target of neutralizing antibodies (nAbs) towards COVID-19 therapy (Corti et al., 2021; Kumar et al., 2021; Kyriakidis et al., 2021). SARS-CoV-2 nAbs provide an attractive alternate strategy for immediate therapy or prophylaxis to COVID-19, especially in immunocompromised patients, unvaccinated, vaccine-hesitant patients, and also in situations where vaccines are less effective against a particular variant (Siemieniuk et al., 2021; Boeckel et al., 2022; Gentile and Moriello, 2022; Gupta et al., 2022). Therapeutic nAbs primarily function by blocking the entry of the virus into the host cells, and perhaps also facilitate the elimination of infected host cells by Fc-mediated effector functions and reducing viral load *in vivo* (Kumar et al., 2021). When administered as prophylaxis or during the early stage of a natural infection, nAbs have been reported to reduce the incidence of hospitalizations and mortality (Kumar et al., 2021). NAb therapy is not specified for severe COVID-19 cases requiring hospitalization (Corti et al., 2021). Except a few minor side-effects (e.g. diarrhea) reported in ~1% of patients after infusion of nAb based therapy in COVID-19 individuals, no major side-effects have been observed (Corti et al., 2021; Gupta et al., 2022). Overall, these findings suggest that broadly neutralizing antibodies (bnAbs) based therapies are generally safe and effective for COVID-19 treatment.

## Recent Progress in Broadly Neutralizing Antibodies Against SARS-CoV-2 Variants and Other Human Coronaviruses

**Figure 1:**
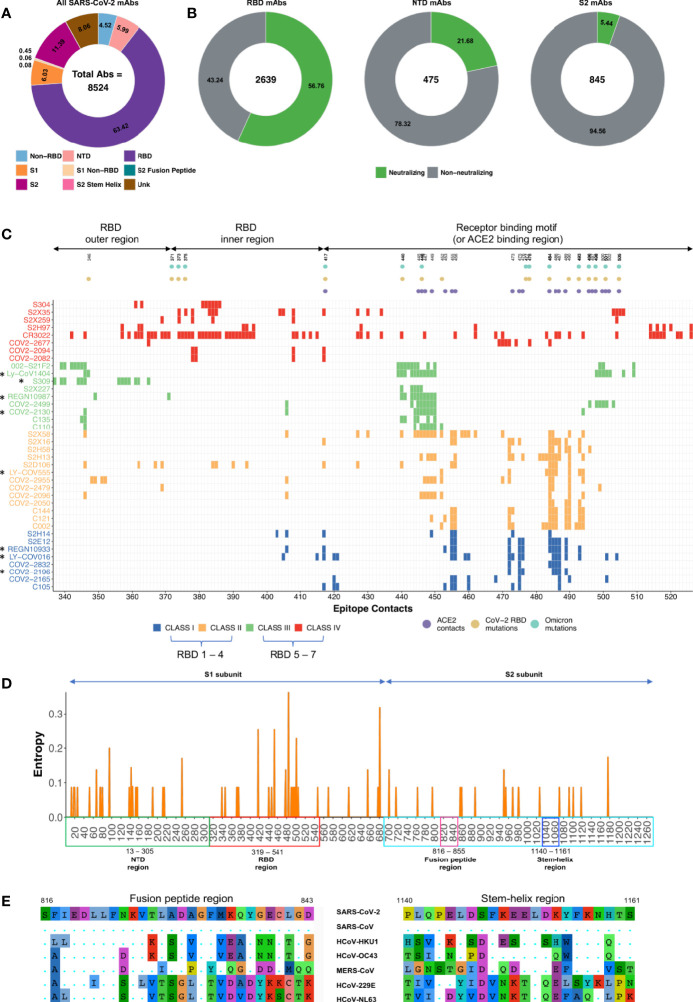
Description of SARS-CoV-2 monoclonal antibodies, their contact epitopes and information on spike conserved regions. **(A)** Distribution of total SARS-CoV-2 mAbs identified so far as documented in CoV-AbDab database and by Wang et al. (Raybould et al., 2021; Wang et al., 2022). **(B)** All mAbs are classified based on the region specificity and distribution of neutralizing (green) vs non-neutralizing (gray) mAbs. Frequency of mAbs targeting the stem-helix region (0.08%) and fusion peptide region (0.06%) are not visible in the plot due to their very low frequency. The mAbs for which neutralization data was unavailable were removed. **(C)** Epitope contacts of RBD specific SARS-CoV-2 mAbs are shown. Targeted NAb epitopes of RBD specific class I to class IV and therapeutic NAbs (marked in asterisks) along with ACE2 contacts, RBD mutations and Omicron mutations are highlighted. Information of contact residues for each mAb shown here was obtained from previously reported studies (Greaney et al., 2022; Kumar et al., 2022; Zhou T. et al., 2022). **(D)** Mutations identified among the key SARS-CoV-2 variants from Alpha to Omicron documented in CoVariants database (Elbe and Buckland-Merrett, 2017) is represented as a Shannon entropy plot. Shannon entropy, is a measure of diversity and hence conservation at each amino acid residue, was calculated using R package Bio3d. The x-axis shows the amino acid residues while the y-axis shows the Shannon entropy value at each residue. The value of Shannon entropy ranges from 0 (no amino acid variation at that position) to 4.32 (all amino acids are equally represented at that position). NTD, RBD, S2 subunit, fusion peptide region and stem-helix region are highlighted in green, red, cyan, pink, and blue respectively. **(E)** Mutations in fusion peptide region and stem-helix region of β-CoVs (SARS-CoV-2, SARS-CoV, HCoV-HKU1, HCoV-OC43 and MERS-CoV) and α-CoVs (HCoV-229E and HCoV-NL63) are shown.

Presently, 8524 SARS-CoV-2 specific monoclonal antibodies (mAbs) have been reported (Raybould et al., 2021; Wang et al., 2022) (**Figure 1A**). A large proportion of mAbs target RBD, N-terminal domain (NTD), and S2 domains of the SARS-CoV-2 spike protein (Wang et al., 2022). A total of 2639/5406 (RBD-specific), 475/511 (NTD-specific), and 845/976 (S2-specific) mAbs have been tested in the SARS-CoV-2 neutralization assay. Of these, 56.76% (1498/2639), 21.68% (103/475), 5.44% (46/845) are neutralizing mAbs targeting RBD, NTD and S2 respectively (**Figure 1B**) (Raybould et al., 2021). The global consortium study has underscored four major classes (I, II, III, and IV) of potent RBD-specific nAbs (Barnes et al., 2020) as RBD1-7 types (Hastie et al., 2021). RBD class I-II or RBD1-4 specific nAbs overlap with ACE2 binding regions and thus as expected are not very effective against major variants of concern (VOCs) and variants of interest (VOIs) where mutations have occurred in the ACE2 binding contacts (Hastie et al., 2021) in the receptor binding motif (RBM) region of the RBD. In contrast, nAbs belonging to class III-IV or RBD5-7 targeting relatively conserved least mutated outer/inner RBD regions thus far effectively neutralizing most VOCs and VOIs (Hastie et al., 2021; Greaney et al., 2022) (**Figure 1C**). Before the emergence of Omicron VOC, RBM directed mAbs with gene usage VH1-58 were among the most potent mAbs (e.g. S2E12, COV2-2196) which were highly effective against all SARS-CoV-2 variants (Tzou et al., 2020). Eight nAbs targeting the RBD region were approved by the US-FDA under emergency use authorization for COVID-19 treatment (EUA) (Kumar et al., 2021; Corti et al., 2021). Among these, only Sotrovimab (S309) neutralizes Omicron (BA.1) variant but poorly neutralizes the BA.2 variant (Cao et al., 2022). Other therapeutic nAbs have been ineffective towards Omicron, mainly due to the presence of extensive mutations in their epitopes, remodeling of the antigenic surface of the spike trimer and dominance of RBD closed state of Omicron spike protein (**Figure 1C**) (Cao et al., 2022; Gobeil et al., 2022; Guo et al., 2022; Zhang et al., 2022). Therefore, for the treatment of Omicron infected patients, US-FDA has provided emergency use authorization (EUA) to a class III or RBD-5 targeting bnAb Bebtelovimab (or LyCoV1404) in Feb 2022 that maintains its potency and neutralizes all SARS-CoV-2 VOCs (Westendorf et al., 2022). Currently, majority of the RBD class III mAbs (Bebtelovimab, 002-S21F2 and Sotrovimab) binding the outer RBD region have shown highest effectiveness against Omicron variants (Kumar et al., 2022; Zhou T. et al., 2022). Interestingly, though Omicron mutations are present in the epitope regions of Bebtelovimab and 002-S21F2 SARS-CoV-2 bnAbs, no loss in potency have been reported (Kumar et al., 2022; Westendorf et al., 2022). This has been explained in their structural studies showing that Omicron mutations in their targeted regions are either favoring the binding by making favorable interaction or not a part of epitope residues targeted by Bebtelovimab and 002-S21F2 bnAbs (Kumar et al., 2022; Zhou T. et al., 2022). This suggests that understanding the structural basis of bnAbs mediated neutralization mechanism of immune evading viruses can provide blueprints to guide structural-based therapeutics and vaccine design by employing affinity maturation through directed evolution, CDRH3 swapping and Reverse Vaccinology 2.0 approaches, respectively (Burton, 2017; Kadam et al., 2017; Traboulsi et al., 2021; Zhao et al., 2021; Zupancic et al., 2021). Moreover, RBD mAbs from hybrid immune individuals dominantly target class III and IV epitopes with potent broad sarbecoviruses neutralizing potential (He et al., 2022; Muecksch et al., 2022). These SARS-CoV-2 bnAbs exhibit unique immunogenetic features like presence of ‘YYDRxG’ motif in their CDRH3 region, enrichment of IGHV3-30, IGHV1-46, IGHV1-69 germline V-genes, IGHD2-15 and IGHD3-22 germline D-genes, moderately higher somatic hypermutation (SHM) >5% (He et al., 2022; Muecksch et al., 2022). On the other hand, nAbs targeting NTD have not been ideal for clinical use development due to recurring mutations in the epitopes they target, resulting in lower effectiveness against the majority of the VOCs and VOIs (Hastie et al., 2021). By contrast, few RBD conserved region-directed nAbs (e.g. S2X324, S2K146, S2X259 and S2H97) have shown broad *in vitro* cross-neutralization potential and *in vivo* protective efficacy at low doses against sarbecoviruses (Starr et al., 2021; Tortorici et al., 2021; Park et al., 2022; Park et al., 2022). Interestingly, a rare class of nAbs (e.g. S2P6, CV3-25, and CC40.8) targeting the pan-β-CoVs conserved hydrophobic regions (stem-helix region) involved in the fusion machinery neutralizes not only all SARS-CoV-2 variants but also pan-β-CoVs (Pinto et al., 2021; Zhou et al., 2022b; Hurlburt et al., 2022). Though these stem-helix specific nAbs neutralize pan-β-CoVs at a higher concentration (IC_50_ >1μg/ml), these bnAbs have shown *in vivo* protective efficacy at low-dose against all 3 major human infecting coronaviruses that include SARS-CoV, MERS-CoV and SARS-CoV-2 (Zhou et al., 2022a) suggesting antibody effector functions *via* their Fc-region may be playing a role in virus clearance. Lastly, clinical evaluation is awaited for recently identified a new class of conserved fusion peptide-directed antibodies (COV44-62 and COV44-79) with the broadest neutralization potential against both α-CoVs and β-CoVs (Dacon et al., 2022).

## SARS-CoV-2 Broadly Neutralizing Antibodies Target Conserved Regions on the Spike Protein

Viral variants can arise due to increasing and persistent immune pressure from both B and T cell compartments on a particular region of the virus (Harvey et al., 2021). Several mutations arise during the normal course of viral replication and many of these mutations may not affect the virus, or some may even be harmful to the virus and thus go unnoticed (Upadhyay et al., 2021). However, during this process of viral propagation/replication, certain mutations can result in increased viral fitness. For example, the generation of viral mutants acquires the ability to escape host immune responses (Harvey et al., 2021; Upadhyay et al., 2021). Mutations reported in the SARS-CoV-2 VOCs and VOIs revealed that these mutations primarily occur in the NTD region, ACE2 contact regions of the RBD, and furin cleavage site region of the S2 domain with few minor exceptions to other residues of S1 and S2 domains (**Figure 1D**) (Tzou et al., 2020; Harvey et al., 2021; Upadhyay et al., 2021). One plausible reason behind this pattern might be the high immune selection pressure elicited by nAbs targeting these key regions during natural infection in the host or vaccinated individuals (Garcia-Beltran et al., 2021). In contrast, no mutation is reported in the broadly conserved fusion peptide and stem-helix regions of the S2 domain of SARS-CoV-2 VOCs/VOIs (Pinto et al., 2021; Dacon et al., 2022; Hurlburt et al., 2022; Zhou et al., 2022b) (**Figure 1E**) which are also recognized by the host’s immune system during natural infection (Wang et al., 2021). The sequence of the fusion peptide region is identical in all SARS-CoV-2 variants and highly conserved across human coronaviruses (Dacon et al., 2022) (**Figure 1E**). NAbs targeting these two regions block fusion machinery by preventing S2 domain refolding from the pre- to the post-fusion state. Limited exposure of these regions to the host immune response or critical requirement of their conserved sequences may be responsible for low antibody-mediated immune pressure, hence limiting the occurrence of mutations in these epitopes (Harvey et al., 2021; Pinto et al., 2021; Upadhyay et al., 2021). Further, both stem-helix and fusion peptide directed nAbs are rarely elicited in convalescent individuals, but their frequencies are higher, with enhanced affinity and breadth in vaccinated individuals who were previously infected (Pinto et al., 2021; Zhou et al., 2022a). For example, Zhou et al. screened 247 SARS-CoV-2 specific mAbs and found that 16% (40/247) mAbs exhibited cross-reactive binding to stem-helix peptides from at least two different β-CoVs (Zhou et al., 2022a). Dacon et. al., screening identified only 2% of 211 mAbs showed broad reactivity to at least two other non-SARS β-CoVs, suggesting that frequency of fusion peptide targeted mAbs are lower than stem-helix mAbs (Dacon et al., 2022). Overall, these studies suggest that nAb cocktails that include relatively conserved least mutated RBD regions, fusion peptides, and stem-helix regions targeting nAbs could be effective against evolving SARS-CoV-2 variants and human coronavirus outbreaks that may arise in the future.

## Conclusions and Future perspectives

Lessons learned with the emergence of Omicron and its sublineages there is an anticipation that more evolving variants may emerge in the future and there is also a potential for future coronavirus outbreaks. This provides an impetus for urgent discovery and development of exceptionally potent bnAbs-based cocktails comprising bnAbs targeting distinct conserved epitopes on spike protein which are effective against pan-SARS-CoV-2 variants and a broad range of human coronaviruses. RBD-specific bnAbs targeting the ACE2 binding region could also be effective against highly mutated variants when an affinity matured bnAb of much higher affinity with RBD than the host ACE2 receptor is developed e.g. a SARS-CoV-2 specific multabody neutralized the virus at extremely low picomolar (pM) concentration (Rujas et al., 2021). Recently, an affinity matured RBD specific nAb CAB-A17 targeting the ACE2 binding region have been shown to neutralize highly mutated Omicron without losing the potency (Sheward et al., 2022). Interestingly, two RBM specific ACE2 blocking SARS-CoV-2 bnAbs S2K146 and S2X324 with cross-reactivity to other SARS-related viruses have been reported to potently neutralize broad SARS related viruses and Omicron variants (Park et al., 2022; Park et al., 2022). Considering all that is known about the structure-function relationship between antibodies and SARS-CoV-2, the following strategies could be effective to combat highly mutated SARS-CoV-2 variants and any coronavirus outbreak(s) in the future: (1) by developing more potent bnAbs targeting RBD conserved regions (e.g. class III/IV or RBD5-7), fusion peptide and stem-helix regions. (2) by enhancing the potency of existing bnAbs, especially directed against the fusion peptide and stem-helix regions, through engineering them with a directed evolution or other antibody engineering approaches, e.g. a non-nAb CR3022 was engineered to a nAb using the directed evolution approach (Zhao et al., 2021). (3) by generating multi-specific bnAbs targeting distinct conserved regions of the RBD, fusion-peptide and stem-helix regions e.g. Bi-specific, Tri-specific, and Multabody approaches as these have shown higher potency, affinity, and breadth against SARS-CoV-2 variants and human CoVs (De Gasparo et al., 2021; Rujas et al., 2021). (4) understanding the structural/molecular antibody-antigen interaction mechanisms of bnAbs effective against pan-SARS-CoV-2 variants, α-CoVs and β-CoVs are critically important for the development of effective therapeutic bnAb cocktails and structure-guided universal vaccine design. (5) by designing effective vaccine candidates based on epitopes targeted by RBD class III bnAbs (Bebtelovimab, 002-S21F2 and Sotrovimab) as these bnAbs can overcome the mutational plasticity of the spike protein in Omicron variants. Structural footprints of these highly potent Omicron neutralizing bnAbs can potentially guide rational vaccine design focused to steer B cells to elicit such bnAbs. Epitope focused vaccines to elicit HIV-1 bnAbs VRC01 and PGT121 have been successfully developed which are currently in clinical trials (Jardine et al., 2015; Steichen et al., 2016). Moreover, *in-vivo* half-life of the potential bnAbs can be increased by incorporating LS-mutation in the Fc domain (Zalevsky et al., 2010; Ko et al., 2014). Additionally, cost-effective bnAbs with persistent *in-vivo* expression can be developed through DNA, mRNA and viral vector-based therapeutics (Patel et al., 2020; Gardner, 2020; Deal et al., 2021). A combination of any of these approaches could potentially dilute immune pressure across the conserved regions of the spike and not specifically target the ACE2 binding region that encourages the generation of viral escape mutants. These efforts will also empower the rapid selection of therapeutic bnAb-based cocktails to alleviate the destructive effects of evolving SARS-CoV-2 variants and outbreak(s) of any human coronavirus in the future.

## Author Contributions

Conceptualization: SK and AC; Writing original draft: SK, PB, VS, and AC; Reviewing and editing of the manuscript: SK, PB, and AC. All authors contributed to the article and approved the submitted version.

## Funding

SK is supported by the DBT/Wellcome Trust India Alliance Early Career Fellowship grant IA/E/18/1/504307.

## Conflict of Interest

The authors declare that the research was conducted in the absence of any commercial or financial relationships that could be construed as a potential conflict of interest.

## Publisher’s Note

All claims expressed in this article are solely those of the authors and do not necessarily represent those of their affiliated organizations, or those of the publisher, the editors and the reviewers. Any product that may be evaluated in this article, or claim that may be made by its manufacturer, is not guaranteed or endorsed by the publisher.
